# Incidence and Time Point of Sepsis Detection as Related to Different Sepsis Definitions in Severely Burned Patients and Their Accompanying Time Course of Pro-Inflammatory Biomarkers

**DOI:** 10.3390/jpm11080701

**Published:** 2021-07-23

**Authors:** Pia Niggemann, Daniel Rittirsch, Philipp Karl Buehler, Riccardo Schweizer, Pietro Giovanoli, Theresia Reding, Rolf Graf, Jan Alexander Plock, Holger Jan Klein

**Affiliations:** 1 Department of Plastic Surgery and Hand Surgery, Burn Center, University Hospital Zurich, 8091 Zurich, Switzerland; Pia.Niggemann@usz.ch (P.N.); Daniel.Rittirsch@usz.ch (D.R.); Riccardo.Schweizer@usz.ch (R.S.); Pietro.Giovanoli@usz.ch (P.G.); 2 Institute of Intensive Care Medicine, University Hospital Zurich, 8091 Zurich, Switzerland; Philipp.Buehler@usz.ch; 3 Regenerative and Reconstructive Plastic Surgery Research Laboratory, University of Zurich, 8091 Zurich, Switzerland; 4 Pancreas Research Laboratory, Department of Visceral Surgery & Transplantation, University Hospital Zurich, 8091 Zurich, Switzerland; theresia.reding.graf@usz.ch (T.R.); rolf.graf@usz.ch (R.G.); 5 Department of Plastic Surgery and Hand Surgery, Cantonal Hospital Aarau, 5001 Aarau, Switzerland; jan.plock@ksa.ch

**Keywords:** burns, sepsis, pro-inflammatory biomarkers

## Abstract

Background: Diagnosis of sepsis in burn patients remains difficult for various reasons. One major problem is the definition of sepsis itself. Therefore, previous and current sepsis definitions are a matter of ongoing validation, but a well-defined consensus on which clinical and laboratory parameters to incorporate in such a definition is lacking. The aim of the present study was to compare the incidence and time-related occurrence of septic events according to different definitions as well as their accompanying time course of pro-inflammatory biomarkers. Methods: Across the first 14 days after admission, the incidence and time point of sepsis according to three different definitions (Sepsis-3, Sepsis American Burns Association [ABA] 2007, Sepsis Zurich Burn Center) were assessed on a daily basis in adult burn patients with total body surface area (TBSA) ≥15% admitted to the Zurich Burn Center between May 2015 and October 2018. In order to investigate how well daily drawn proinflammatory biomarkers (white blood cells (WBCs), C-reactive protein (CRP), procalcitonin (PCT), and novel pancreatic stone protein (PSP)) reflect the progression of sepsis depending on its type of definition, a longitudinal mixed model analysis was performed across the first 14 days for septic and non-septic patients. Additionally, the relative increase of biomarker levels 24, 48, and 72 h prior to a septic event was analyzed for each definition used. Results: In our cohort of 90 severely burned patients, Sepsis-3 identified 46 patients (51.1%) as septic, while ABA 2007 and the Zurich Burn Center definition counted 33 patients (36.7%) and 24 patients (26.6%), respectively. Sepsis-3 detected sepsis about 1 day earlier than Sepsis ABA 2007 (*p* < 0.001) and about 0.5 days earlier than Sepsis Zurich Burn Center (*p* = 0.04). The course of pro-inflammatory biomarkers was largely unaffected by the type of sepsis definition. Irrespective of the sepsis definition, PSP was the only marker to demonstrate a highly significant interaction between time and group (sepsis versus no sepsis) (*p* < 0.001) with a 3.3–5.5-fold increase within 72 h before the event of sepsis, whereas CRP, PCT, and WBC showed only mild undulations. Conclusions: Despite the ongoing dilemma of how to define sepsis in burn patients, a continually calculated SOFA score as used in Sepsis-3 is advantageous to early identify a patient’s detrimental progression to sepsis. Inclusion of biomarkers, such as PSP, may help support the burn specialist’s diagnosis of sepsis and could improve the diagnostic performance of current and future definitions in burn patients.

## 1. Introduction

Despite an increasing incidence of septic courses in severely burned patients, their survival has improved thanks to sophisticated burn wound care, enhancements of intravenous fluid administration and infection control measurements, as well as nutritional support [1]. Nevertheless, uncontrolled sepsis still represents the major cause of death in cases of burn injury [2]. The key limiting factor for the poor clinical outcome of septic patients —even under best possible care—remains the lack of reliable diagnostic tools, which promptly recognize critical events with subsequent initiation of targeted intensified care [3,4].

On the one hand, the greatest challenge to identify a patient’s septic progression lies within the sepsis definition itself, both in clinical routine and research settings. This may lead to the discrepancy that patients are diagnosed with sepsis in accordance with one definition, whereas another definition does not yet—if at all—consider those patients septic. Back in 1991, the first internationally accepted definition of sepsis (Sepsis-1) was introduced. It was based on the systemic inflammatory response syndrome (SIRS) as a reaction to infection [5]. The International Sepsis Definition Conference revised the definition in 2001 and presented Sepsis-2, which was also based on SIRS with a complementary infection [6]. Despite its additional signs and symptoms, no evidence was found to support a wide-ranging shift to Sepsis-2 [7]. In 2007, members of the American Burn Association (ABA) revised the Sepsis-1 definition for burn patients and accounted for the accompanying baseline SIRS, which is regularly observed in burns with TBSA > 15%, by adding elevated cut-off values for heart and respiratory rates, feeding and glucose intolerance, and thrombocytopenia [8]. In 2016, experts of the Society of Critical Care Medicine and the European Society of Intensive Care Medicine introduced the Third International Consensus Definition (Sepsis-3), in which sepsis is described as a life-threatening organ dysfunction caused by a dysregulated host response to infection [9]. This was the first definition that completely left the SIRS concept, but instead used the sequential organ failure assessment (SOFA) score in the context of infection. The usability of the Sepsis-3 definition in burn patients is a matter of current debate and ongoing validation. Recent findings regard the Sepsis-3 definition to be superior by having 85% sensitivity over the ABA sepsis definition with its 60% sensitivity [10,11].

On the other hand, especially in burns, where early sepsis diagnosis and initiation of therapy is key to survival, clinicians include pro-inflammatory biomarkers like C-reactive protein (CRP) or procalcitonin (PCT) into their decision-making process. However, such biomarkers are not taken into account in previous and current sepsis definitions, with few exceptions. Only Sepsis-1 includes the white blood cell count (WBC), while Sepsis ABA 2007 and Sepsis-3 include the thrombocyte count as a “pro-inflammatory correlate”. Additionally, these canonical biomarkers often fail to uncover infectious events at an incipient stage of the disease as their alterations are prone to interfere with further stimuli such as trauma severity (i.e., total burned surface area (TBSA) in burns), repetitive surgical interventions, and concomitant conditions like inhalation injury [12,13,14]. Recently, evidence on the usefulness of pancreatic stone protein (PSP) as an accurate diagnostic and prognostic marker in critically ill patients is accumulating [15,16,17]. Originally described as a protein constitutively secreted by pancreatic acinar cells to inhibit growth and nucleation of calcium carbonate crystals, insights from more recent studies suggest PSP as acute phase protein activating neutrophil granulocytes in the early phase of infection. Our group recently published data on PSP’s excellent predictive power to detect sepsis and septic shock (Sepsis-3) in burn patients—outperforming PCT and CRP by its steeper and earlier rise prior to the critical onset of sepsis [18].

Against this background, we sought to compare the incidence and time-related occurrence of septic events between three different sepsis definitions in a cohort of 90 severely burned patients over the first 14 days after admission to the Zurich Burn Center. Additionally, our goal was to assess how well pro-inflammatory biomarkers (WBC, CRP, PCT, PSP) reflect the event of sepsis depending on its type of definition.

## 2. Materials and Methods

### 2.1. Ethics Approval

Ethics approval was obtained from the Ethics committee of the University of Zurich, Switzerland on 20 April 2015 (KEK-ZH-No: 2014-0631).

### 2.2. Participants

Patients with burns ≥15% TBSA admitted to our burn center from May 2015 to October 2018 were asked to participate. All patients were provided with comprehensive oral and written information on the present study and asked to sign the informed consent. If a patient was unable to give consent owing to the extent of injury, close relatives were asked for the patient’s presumed intention. Patients with TBSA <15%, aged <18 years, with infection at admission, or burn injuries older than 6 h and immunosuppressive medication were excluded.

The sample size calculation refers to the biomarker part of the present study focusing on the interaction between time (14 time points) and status of sepsis (i.e., two groups), as bedside clinicians are interested in the change over time of a biomarker to detect a patient’s deterioration. Previous findings on PSP in infected patients performed by our group revealed an effect size Cohen’s f = 0.2 (0.10, 0.25, and 0.40 represent small, medium, and large effect sizes, respectively). Owing to the burn patients’ inherent baseline inflammation potentially interfering with biomarker levels in infected/septic patients, we chose an even lower, conservative effect size of about 0.1–0.15 for the present study. Based on these pre-study considerations, a priori sample size estimation was performed using GPOWER 3.1, resulting in 82 patients required (given α = 0.05, power = 0.9, effect size = 0.1, number of measurements = 14, correlation among measurements = 0.5) [19].

### 2.3. Measurement of Serum Plasma Biomarker Concentration

Blood samples for measurement of conventional inflammatory biomarkers (CRP, WBCs, and PCT) and PSP were drawn daily starting on the day of the patient’s admission to our burn center. Leukocytes were counted by flow cytometry, while CRP and PCT levels were determined by routine measurements of the Department of Clinical Chemistry, University Hospital Zurich. While the aforementioned markers were directly measured as routine parameters, serum samples for determination of PSP were stored at −80 °C for subsequent analysis. The concentration of PSP/REG Iα was measured with an isoform specific ELISA, which was established in our laboratory [20,21]. Details on measurement have been published previously [18].

### 2.4. Patient-Related Data

The patients’ demographic (gender, age, body mass index (BMI)) and trauma-related (TBSA, mechanism of injury, inhalation injury) data were recorded, and clinical parameters were collected for 14 consecutive days. The clinical parameters included blood counts, electrolytes, inflammatory markers (CRP, PCT, PSP), liver, kidney and pancreas function, as well as vital signs (ventilation, mental status, heart frequency, and blood pressure). Samples and measurements were usually taken at 06:00 daily. All treating physicians were blinded to PSP results, whereas they were aware of CRP, PCT, and WBCs.

### 2.5. Definition of Nosocomial Infections

Using the Centers for Disease Control and Prevention (CDC) definition for hospital acquired infections, we distinguished between pneumonia, bacteremia, central-line, urinary tract, and wound infection [22].

### 2.6. Definitions of Sepsis

- American Burns Association 2007 (Sepsis ABA 2007)

Temperature >39 °C or <36.5 °C, progressive tachycardia >110 bpm, progressive tachypnea >25 bpm (if ventilated >12 L/min), thrombocytopenia <100,000/μL, hyperglycemia (absence of diabetes) plasma glucose >200 mg/dL or >7 units of insulin/hour intravenous, or significant resistance (increase >25%) to insulin. Patients meeting ≥3 criteria coupled with a documented infection should be seen as septic [23,24].

- Sepsis-3

The Third International Consensus Definition for Sepsis and Septic Shock (Sepsis-3) is defined as life-threatening organ dysfunction caused by a dysregulated host response to a suspected or confirmed infection, objectively described as increase of sequential organ failure assessment (SOFA) score ≥2 points in the presence of a confirmed or suspected infection. Patients with septic shock meet the criteria of sepsis with persistent hypotension requiring vasopressors to maintain MAP ≥65 mmHg and lactate level ≥2 mmol/L despite adequate volume resuscitation [9]. An increase in the SOFA score ≥2 was evaluated daily using a rolling baseline.

- Zurich Burn Center

Like many burn centers around the world (e.g., [10]), the Zurich Burn Center follows a prospective clinical assessment on an individual basis including the SIRS-criteria as adapted for burn patients by the ABA 2007 definition, an increase of the SOFA score ≥2 on a rolling basis and alterations of pro-inflammatory biomarkers (CRP, WBC, PCT). There are no fixed cut-off values, but rather alterations over time of clinical and laboratory markers that support our bedside clinicians when diagnosing sepsis.

The status of sepsis according to the ABA 2007 and Sepsis-3 definitions was retrospectively ascertained on a daily basis corresponding to the time point of biomarker analysis (around 07:00 daily). Accordingly, the date of sampling that subsequently turned out positive was taken as the time point of infection or sepsis. Sampling was performed at the discretion of the clinician. Sepsis Zurich Burn Center was determined prospectively by bedside clinicians and extracted for the present retrospective study.

### 2.7. Statistical Analysis

Discrete values are expressed as counts with percentages, while continuous variables are presented as mean ± standard deviation (SD) or median with interquartile range (IQR) as appropriate. Ordinal and continuous baseline characteristics were tested with the Kruskal–Wallis test across the three sepsis groups. Wilcoxon matched pair signed rank test was used to test differences between the detected onsets of the three sepsis definitions. Biomarker time courses were compared between groups (sepsis vs. no sepsis) using a linear mixed effects regression model with random intercepts focusing on daily main-effects as well as the interaction between time and group (sepsis vs. no sepsis). All tests were two tailed; *p* < 0.05 was considered significant. To account for multiple testing in the main effect analysis of the linear mixed effects regression model, Bonferroni correction was applied. To account for time-dependency of the status of sepsis, an event-related analysis was performed by re-arranging the individual biomarker time courses according to the date of the septic event. The ratio between biomarker levels at the event of sepsis and 24, 48, and 72 h before the event of sepsis was calculated and compared across the three mentioned sepsis definitions. Data were analyzed using Jamovi (The jamovi project (2019), jamovi (Version 1.0.5)) and GraphPad Prism version 6.00 for Macintosh (GraphPad Software, La Jolla, CA, USA).

## 3. Results

### 3.1. Baseline Characteristics as Related to Different Sepsis Definitions

**Figure 1:**
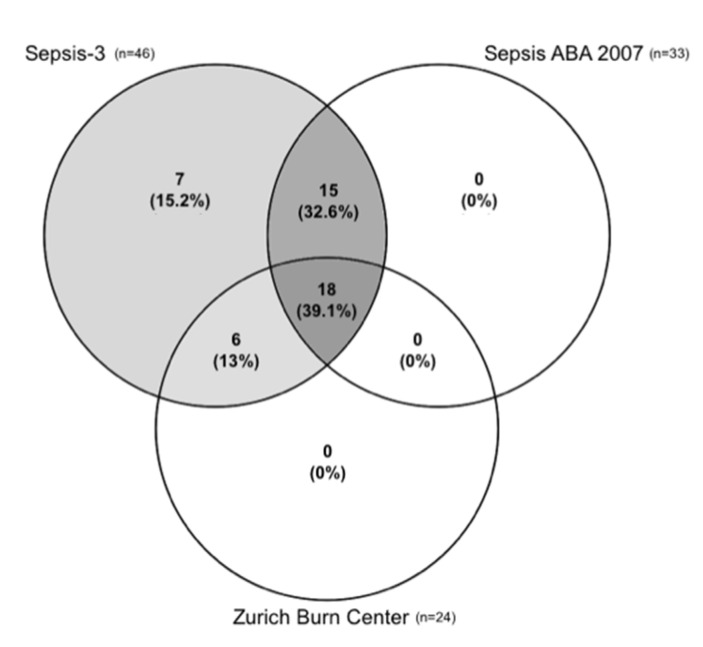
Venn diagram depicting the overlapping quantities of the patients meeting the criteria for the three different sepsis definitions. Sepsis was diagnosed across all definitions in 18 patients (20%). Thirty-three patients (37%) fulfilled the conditions of both Sepsis-3 and Sepsis American Burn Association (ABA) 2007. Sepsis-3 and Sepsis Zurich Burn Center had an overlap of 24 (27%) patients.

In total, 90 severely burned patients with TBSA ≥15% were included with a mean age of 48.5 ± 18.8 years and a median burn size of 32% (IQR 21). Sepsis-3 detected sepsis in 46 patients (51.1%), while the ABA 2007 and the Zurich Burn Center definition counted 33 patients (36.7%) and 24 patients (26.6%), respectively. The Venn diagram depicted in Figure 1 shows the overlapping quantities of the patients meeting the criteria for the three sepsis definitions. Sepsis was diagnosed across all definitions in 18 patients (20%). Thirty-three patients (37%) fulfilled the conditions of both Sepsis-3 and Sepsis ABA 2007. Sepsis-3 and Sepsis Zurich Burn Center had an overlap of 24 (27%) patients. Accordingly, Sepsis-3 encompassed all patients of Sepsis ABA 2007 and Sepsis Zurich Burn Center. Demographic and injury-related characteristics are shown in Table 1. Median age was not significantly different between the three groups (*p* = 0.38). Median TBSA was higher in the Zurich Burn Center Sepsis group than in the other two, but lacked significance across all three groups (*p* = 0.28). Likewise, the groups did not differ with regard to their ABSI and Baux score (*p*_ABSI_ = 0.55; *p*_Baux_ = 0.66). Of the 90 patients included, 14 died during the investigated 14 days. Ten patients died of reasons other than infection or sepsis. Four patients died within the Sepsis ABA 2007/Sepsis-3 group and three patients died within the Sepsis Zurich Burn Center group (+one patient who died of simple infection).

**Figure 2:**
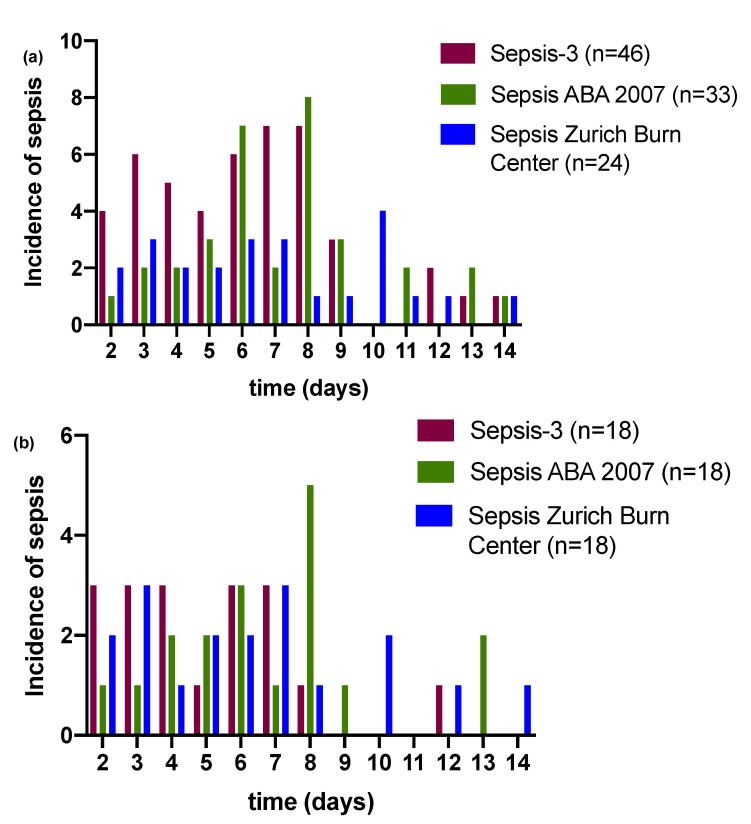
(**a**) Number of patients diagnosed with sepsis by the three definitions on a time scale across 14 days. The average (median) day of sepsis detection was 6 (interquartile range (IQR) 4) in Sepsis-3, 7 (IQR 3) in Sepsis ABA 2007, and 6.5 (IQR 6.0) in Sepsis Zurich Burn Center. (**b**) Sepsis incidence of the common overlap of septic patients across all three definitions (*n* = 18).

Figure 2a depicts the number of patients diagnosed with sepsis by the three definitions on a time scale across 14 days. The average (median) day of sepsis detection was 6 (IQR 4) in Sepsis-3, 7 (IQR 3) in Sepsis ABA 2007, and 6.5 (IQR 6) in Sepsis Zurich Burn Center. Figure 2b shows the incidence of the common overlap of septic patients across all three definitions (*n* = 18). For the common overlap, the median time point of sepsis detection was 4.5 (IQR 4) for Sepsis-3, 6.5 (IQR 3.25) for sepsis ABA 2007, and 6 (IQR 5.5) for Sepsis Zurich Burn Center. Wilcoxon matched pair signed rank test showed that Sepsis-3 detected septic patients earlier than Sepsis ABA 2007 with a median difference of 1 day (*p* < 0.001). Likewise, Sepsis-3 detected sepsis 0.5 days earlier than Sepsis Zurich Burn Center (*p* = 0.04), while there was no significant difference between Sepsis ABA 2007 and Sepsis Zurich Burn Center (*p* = 0.24).

### 3.2. Biomarker Time Course as Related to Different Sepsis Definitions

**Figure 3:**
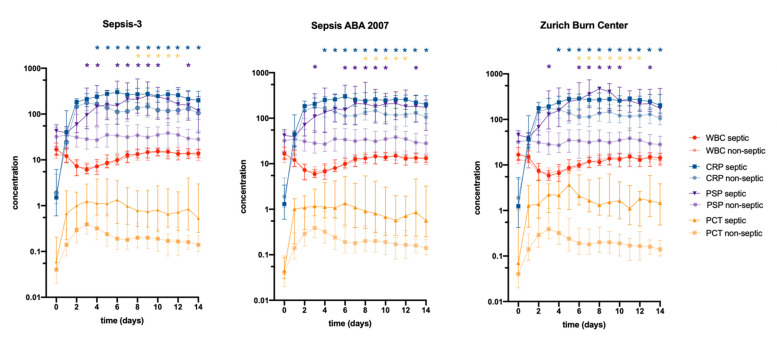
Biomarker time course over 14 days after admission (day 0) stratified for septic and non-septic patients. * Indicates significance (*p* < 0.003) after Bonferroni correction. Note the strong interaction between time and group for pancreatic stone protein (PSP) levels with septic patients having not only higher absolute values, but also demonstrating a crucial increase over time as opposed to non-septic patients.

Figure 3 depicts the time course of WBC, CRP, PCT, and PSP over 14 days according to the different sepsis definitions for septic and non-septic patients. WBC serum values demonstrated a remarkable decline after admission with a nearly equal course of septic and non-septic patients across all three definitions. There was no significant difference in WBC levels in any definition during the 14 days. CRP levels rose quickly after admission and were significantly higher in septic patients as compared with non-septic ones from day 4 onward irrespective of the definition. Similarly, PCT serum levels increased after admission in septic as well as in non-septic patients, but significantly varied in their level of concentration at several subsequent time points. For the Sepsis-3 definition, PCT levels were significantly elevated from day 8 to 12; for the Sepsis ABA 2007, from day 8 to 10; and for the Sepsis Zurich Burn Center definition, from day 7 to 9 and 11 to 12. PSP levels were similar in septic versus non-septic patients within the first 48 h after admission, but strikingly increased afterwards for septic patients with significant differences from day 3 ongoing across all sepsis definitions. Sepsis-3 demonstrated significantly higher PSP levels on day 3, 4, 6 to 10, and 13; Sepsis ABA 2007 on day 3 and 6 to 9, and Sepsis Zurich Burn Center from day 3, 7 to 10, and 13. Non-septic patients, however, demonstrated only mild undulations in PSP levels across all definitions. Consequently, only PSP time course demonstrated a significant interaction between time and group (sepsis vs. no sepsis)—irrespective of a specific definition—marking a steeper increase in septic patients as opposed to those with an uneventful course (*p* < 0.001). On the contrary, WBC, CRP, and PCT values revealed a rather paralleled course for the two groups across 14 days.

### 3.3. Biomarker Time Course as Related to The Event of Sepsis

**Figure 4:**
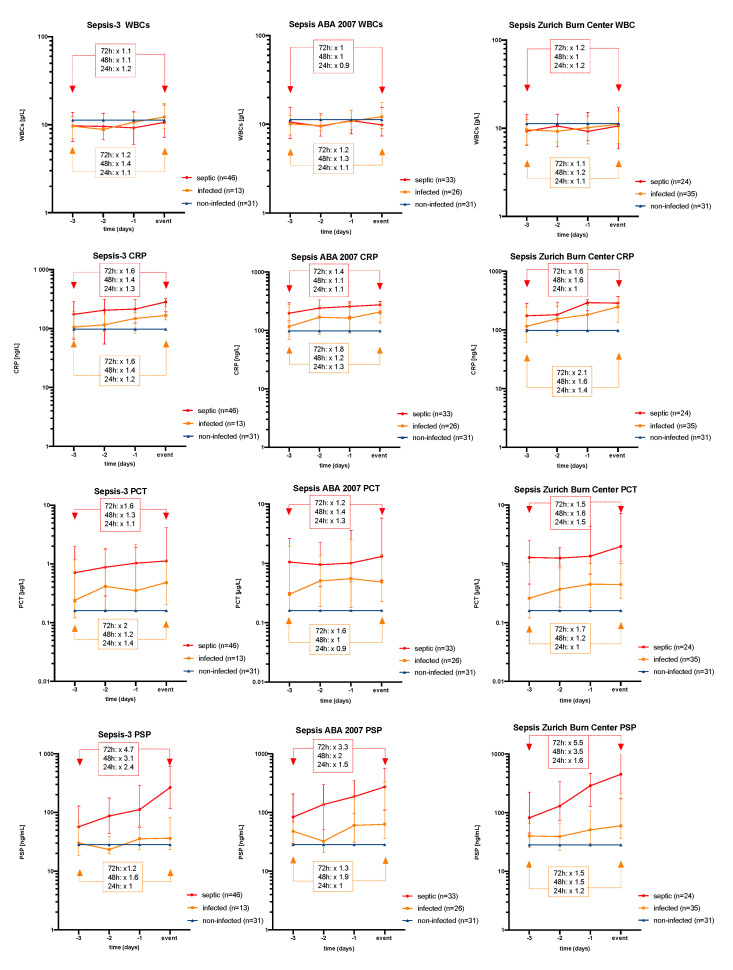
Time course of the biomarkers as related to the event of sepsis. White blood cell (WBC), C-reactive protein (CRP), procalcitonin (PCT), and PSP time courses are shown in single plots for each sepsis definition. Whereas leukocytes, CRP, and PCT showed only mild alterations, PSP consistently demonstrated a steep increase for septic patients up to 72 h before sepsis was diagnosed across all three definitions.

In order to focus on the relative alterations of biomarker concentrations prior to the diagnosis of infection or sepsis, we rearranged the individual biomarker courses according to their time point of the event. The grand median of non-infected patients was used as a reference line. Figure 4 represents the time course of WBC, CRP, PCT, and PSP for each sepsis definition with regard to the event of infection/sepsis. PSP levels were 5-fold higher in the Zurich Burn Center definition, 4.7-fold higher in Sepsis-3, and 3.3-fold higher in the ABA 2007 definition 72 h before the event. PCT and CRP as commonly used sepsis biomarkers were just 1.6-fold higher in the Sepsis-3 and Zurich Burn Center definitions and 1.4-fold higher in the ABA 2007 definition 3 days before sepsis was diagnosed. During the same time period, PSP showed a lower increase in infected patients. WBC, finally, demonstrated only mild undulations prior to the event of sepsis in every definition. Additional ROC-curve analysis of infected versus septic patients 24, 48, and 72 h prior to an infectious/septic event is given for each sepsis definition in  Supplementary Figures S1–S3 and  Supplementary Tables S1–S3.

## 4. Discussion

The present study investigated the incidence and onset of sepsis according to three different definitions as well as the accompanying time course of pro-inflammatory biomarkers (WBC, CRP, PCT, PSP) in a cohort of 90 severely burned patients over 14 days. We found different numbers of septic patients depending on the definition. The Sepsis-3 definition considered more than half of our cohort as septic (51%). ABA 2007 (37%) and the Zurich Burn Center (27%) definition found less patients septic, and thus more closely resembles reported incidences of sepsis with up to 30% throughout the literature [10]. The variability of sepsis incidences in the present study and the discrepancy to the previous studies connotes that the greatest challenge is posed by the sepsis definition itself. While trials before 2016 mainly used the ABA sepsis definition, more recent studies adopted the Sepsis-3 definition, completely leaving the SIRS concept, but considering organ failure in the context of infection as key element. The usability of the Sepsis-3 definition in burns is matter of current debate and validation, but the lacking gold-standard in sepsis diagnosis makes it difficult to evaluate whether one definition is better than the other. Stanojcic et al., for example, used their prospectively documented sepsis criteria, in which the burn team identified sepsis on the basis of various clinical symptoms [10]. They found the Sepsis-3 definition to be superior by having 85% sensitivity over the ABA 2007 sepsis definition with 60% sensitivity. Transferring this approach onto our data with the Sepsis Zurich Burn Center definition as a reference resulted in a sensitivity of 100% for both the Sepsis-3 definition and the ABA 2007 definition. However, these results should be interpreted with caution, as the Sepsis-3 as well as Sepsis ABA 2007 diagnoses of sepsis were made retrospectively with a comprehensive overview of all clinical data, examinations, infection sources, and pathogens available. A reason for the high incidence of septic courses according to Sepsis-3 is that nearly every organ system is stricken by the burn injury, and thus might lead to false positive sepsis detection. Moreover, it is conceivable that the Sepsis-3 definition in the setting of severe burn injury with underlying systemic inflammation is overly sensitive and not specific enough. Accordingly, Yoon et al. found Sepsis-3 not to be superior as compared with earlier sepsis definitions in terms of its diagnostic performance in severely burned patients, but emphasized that the Sepsis-3 criteria including the SOFA score as an indicator of organ dysfunction—for which patients with burns are particularly at risk—is reasonable [25]. Above that, Yoon et al. stated that a SOFA score ≥6 points would be a reliable predictor for sepsis in burns. Similarly, other authors argue that an adaptation of the SOFA score, e.g., to include a severity grading of skin dysfunction, may be appropriate for the burns population [8].

Yan et al. demonstrated that the new Sepsis-3 consensus definition—though not originally developed for burn patients specifically—outperforms the ABA sepsis definition as well as the definition by Mann-Salinas et al. at predicting the onset of sepsis [11]. In the present study, Sepsis-3 diagnosed the onset of sepsis about 1 day earlier than the other definitions. This temporal advance is crucial and beneficial for the initiation of intensified treatment to septic patients, and thus recommends a constant monitoring of the SOFA score to early identify a detrimental progress.

Recent and earlier definitions of sepsis do not include well established pro-inflammatory biomarkers, such as CRP or PCT, although clinicians—and particularly burn specialists—commonly use them to support their decision-making process to diagnose sepsis. Therefore, we further analyzed the course of routinely used pro-inflammatory markers (leukocytes, CRP, and PCT) as well as novel PSP in septic and non-septic patients across the three sepsis definitions. Interestingly, the day-wise main effect analysis (Figure 3) demonstrated nearly equal differences between the two groups across all three sepsis definitions. Besides this unaffectedness of the biomarker course by the type of sepsis definition, PSP revealed higher absolute values for septic patients at several time points after the first 48 h. Despite some days lacking significance between septic and non-septic patients, PSP was the only marker to demonstrate a highly significant interaction between time and group (sepsis versus no sepsis), connoting that septic patients exhibit a crucial increase in PSP serum levels over time as opposed to non-septic patients. These findings have recently been published by our group in detail using the Sepsis-3 definition [14,18,26]. However, results from longitudinal studies on sepsis biomarkers have to be interpreted with caution as the time-dependency of the infectious/septic event is inherently not accounted for. Considering an event (disease) status for an individual as fixed over time is often encountered in such biomarker analyses and has to be rated as a limitation of the present study.

In order to account for time-dependency, we re-arranged the individual biomarker course with regard to the time point of the event (sepsis, infection). The authors’ intention of Figure 4 was to show the relative increase in biomarker levels before the event of sepsis determined by different definitions. In the given scenario of re-arranged individual biomarker courses (setting the event of sepsis at day 0), ROC curve analysis with biomarker sensitivity/specificity is not amenable, as there is no event of “no-sepsis” that could be used as reference. This is why the grand median was presented for non-septic patients as a reference line (blue). Against this background, Figure 4 follows a rather descriptive approach in order to underline PSP’s trending increase prior to the critical event of sepsis. This trend was not observed for leukocytes, CRP, and PCT, displaying only mild alterations up to 72 h before sepsis was diagnosed across all three definitions. Furthermore, as shown in the Supplementary Material, PSP had the highest sensitivity and specificity for septic versus infected patients ranging around 0.8 and 0.8 in the days before the critical event across all definitions. Notably, the Sepsis Zurich Burn Center definition revealed higher absolute values for PCT and PSP around the time point of sepsis when compared with the two other definitions. This might be explained by the fact that the Zurich burn team documented a septic event in that very moment, when the clinical deterioration of a patient was really obvious and PSP and PCT levels were high.

The present study clearly expresses the ongoing dilemma of how to define sepsis in burn patients. Although the usage of the new Sepsis-3 definition might have the tendency to “over-diagnose” sepsis in burn patients, the implementation of a continually calculated SOFA score has its merit in order to monitor a patient’s clinical status and to timely initiate microbiological sampling and targeted sepsis therapy in the case of deterioration. Future studies should thus focus on a potential modification of the SOFA score for burn patients. Combined assessment of the SOFA score and PSP as a robust sepsis biomarker might help identify patients at an incipient stage of deterioration and increase the specificity in burn patients as compared with the SOFA score alone. Together with its high predictive power and its early temporal increase, levels of PSP can readily be measured bedside from a drop of whole blood using a nanofluid based assay (abioSCOPE, Abionic SA, Epalinges, Switzerland) (R.G. is the inventor of an assay covered by patent No: EP 2185937 B2 “METHOD FOR ASSAYING SEPSIS IN HUMANS”, which is owned by the University of Zurich (Zurich, Switzerland)). Without preanalytical work, this device enables physicians to quantify PSP levels at a picomolar range within a few minutes [18]. The use of the abioSCOPE in clinical routine is currently validated in international, multi-center trials. Against this background, the results of the present study need to be confirmed in future clinical trials, since yet, there are some limitations. Given the single-center design of our study, there is no external validation of our data, which has to be addressed in further studies. Along with that, measurement of biomarkers and assessment of clinical parameters were performed only once per 24 h, neglecting potential alterations within these intervals.

## 5. Conclusions

Despite the ongoing dilemma of how to define sepsis in burn patients, a continually calculated SOFA score is advantageous to early identify a patient’s detrimental progression to sepsis. Inclusion of biomarkers, such as PSP, may help support the burn specialist’s diagnosis of sepsis and could improve the diagnostic performance of current and future definitions in burn patients.

## Figures and Tables

**Table 1:** Baseline characteristics in total and according to the main outcome no infection vs. different sepsis definitions. The Kruskal–Wallis test was used for comparison of ordinal and continuous data across the three sepsis groups to rule out a potential bias by age, total body surface area (TBSA), and body mass index (BMI) (ABSI and Baux score) with regard to the subsequent longitudinal analyses.

		Total	No Infection	Sepsis-3	Sepsis ABA 2007	Sepsis Zurich Burn Center	*p*
Number of patients (*n*,%)		90	31 (34.4%)	46 (51.1%)	33 (36.7%)	24 (26.6%)	-
Gender (*n*,%)	female	18 (20%)	6 (33.3%)	9 (50%)	7 (38.9%)	5 (27.8%)	-
	male	72 (80%)	25 (34.7%)	37 (51.4%)	26 (36.1%)	19 (26.4%)	-
Age (years; mean ± SD)		48.5 ± 18.8	50.4 ± 20.6	47.1 ± 18.2	50.2 ± 17.3	43.5 ± 18.2	0.38
TBSA (%; median, IQR)		32 (21)	29.5 (21.6)	35 (24.5)	36 (26)	39.5 (28.8)	0.28
BMI (kg/m^2^; median, IQR)		26.3 (6.8)	27.6 (7.2)	24.8 (6.8)	24.7 (7)	24.7 (7)	0.93
Inhalation injury (*n*, %)		25 (27.8%)	7 (28 %)	16 (64%)	16 (64%)	8 (32%)	-
Total length of stay (days, median, IQR)		28 (38)	18 (24)	46 (61.2)	51 (77)	58 (55.5)	0.70
ICU length of stay (days, median, IQR)		16 (30)	6.5 (11.5)	28 (40)	40 (60)	44 (42.5)	0.81
ABSI (median, IQR)		7.5 (4)	7 (6)	8 (3)	9 (4)	9 (4)	0.55
Baux score (median, IQR)		84 (37.5)	83.5 (48)	86.5 (32)	90 (35)	91.5 (28.5)	0.66
Mortality (*n*, %)		14 (15.5%)	10 (71.4%)	4 (28.6%)	4 (28.6%)	3 (21.4%)	-

## Data Availability

Derived data supporting the findings of this study are available from the corresponding author on request.

## Supplementary Materials

The following are available online at https://www.mdpi.com/article/10.3390/jpm11080701/s1. Figure S1: ROC-curve analysis with AUC for Sepsis-3. AUC of WBC at t−3, −2, −1 is between 0.5 and 0.6. AUC of CRP at t = −3, −2, −1 is between 0.62 and 0.66. AUC of PCT at t = −3, −2, −1 is between 0.59 and 0.65. The best performance is shown by PSP with an AUC of 0.72 (t = −3) 0.88 (t = −2) and 0.85 (t = 0.1) before the event of sepsis. The sensitivity at t = −3 is 0.7, t = −2 is 0.8 and t = −1 is 0.7. The specificity at t = −3 is 0.7, t = −2 is 0.81, t = −1 is 0.9.; Figure S2: ROC−curve analysis with AUC for Sepsis ABA 2007. AUC of WBC at t−3, −2, −1 is between 0.5 and 0.55. AUC of CRP at t−3, −2, −1 is between 0.6 and 0.64. AUC of PCT at t−3, −2, −1 is between 0.6 and 0.62. The best performance is shown by PSP with an AUC of 0.64 (t = −3) 0.74 (t = −2) and 0.72 (t = 0.1) before the event of sepsis. The sensitivity at t = −3 is 0.6, t = −2 is 0.87 and t = −1 is 0.7. The specificity at t = −3 is 0.8, t = −2 is 0.56 and t = −1 is 0.75 v; Figure S3. ROC−analysis with AUC for Sepsis Zurich Burn Centre. AUC of WBC at t = −3, −2, −1 is between 0.47 and 0.55. AUC of CRP at t = −3, −2, −1 is between 0.57 and 0.7. AUC of PCT at t = −3, −2, −1 is between 0.74 and 0.79. The best performance is shown by PSP with an AUC of 0.72 (t = −3) 0.82 (t = −2) and 0.84 (t = 0.1) before the event of sepsis. The sensitivity at t = −3 is 0.57, t = −2 is 0.81 and t = −1 is 0.85. The specificity at t = −3 is 0.83, t = −2 is 0.77 and t = −1 is 0.79, Table S1: ROC-curve analysis for Sepsis-3; Table S2. ROC-curve analysis for Sepsis ABA 2007; Table S3. ROC-curve analysis for Sepsis Zurich Burn Centre.

## Patents

Rolf Graf is the inventor of an assay covered by patent No: EP 2185937 B2 “METHOD FOR ASSAYING SEPSIS IN HUMANS”, which is owned by the University of Zurich (Zurich, Switzerland).
