# Supplementary material for: Incidence and Time Point of Sepsis Detection as Related to Different Sepsis Definitions in Severely Burned Patients and Their Accompanying Time Course of Pro-Inflammatory Biomarkers
Source: J Pers Med. 2021 Jul 23;11(8):701. doi: 10.3390/jpm11080701 (PMC8401386; doi:10.3390/jpm11080701)
Supplement: Supplementary file 1 [file jpm-11-00701-s001.zip › jpm-1273997-supplementary.pdf]

Supplementary material

For the analysis of the progression of biomarkers, we calculated the ratio between measurements at the event of sepsis and 24, 48 and 72 hours before the event of sepsis in septic and infected patients. Noninfected patients had an individual baseline. For predicting whether a patient is septic at the event time (vs only infected patient) using biomarker values at times -3, -2, -1, robust logistic regression models were fitted using the R package robust. Missing data were excluded from the analysis. Therefore, total of 12 logistic regression models were fitted per each sepsis definition (a total of 36 models). For obtaining the Receiver Operator Characteristics (ROC) curve, the R package pROC was used. The optimal probability cut-off values were chosen using Youden's J statistic (Youden, 1950). The AUC and the optimal cut-off, sensitivity, specifity, negative predictive value (NPV) and positive predictive value (PPV) of each model were calculated and presented in Figure S1 to S3 and Table S1 to S3. For this analysis R (R Core Team 2021) was used. (R: A language an environment for statistical computing. R: Foundation for Statistical Computing Vienna, Austria).

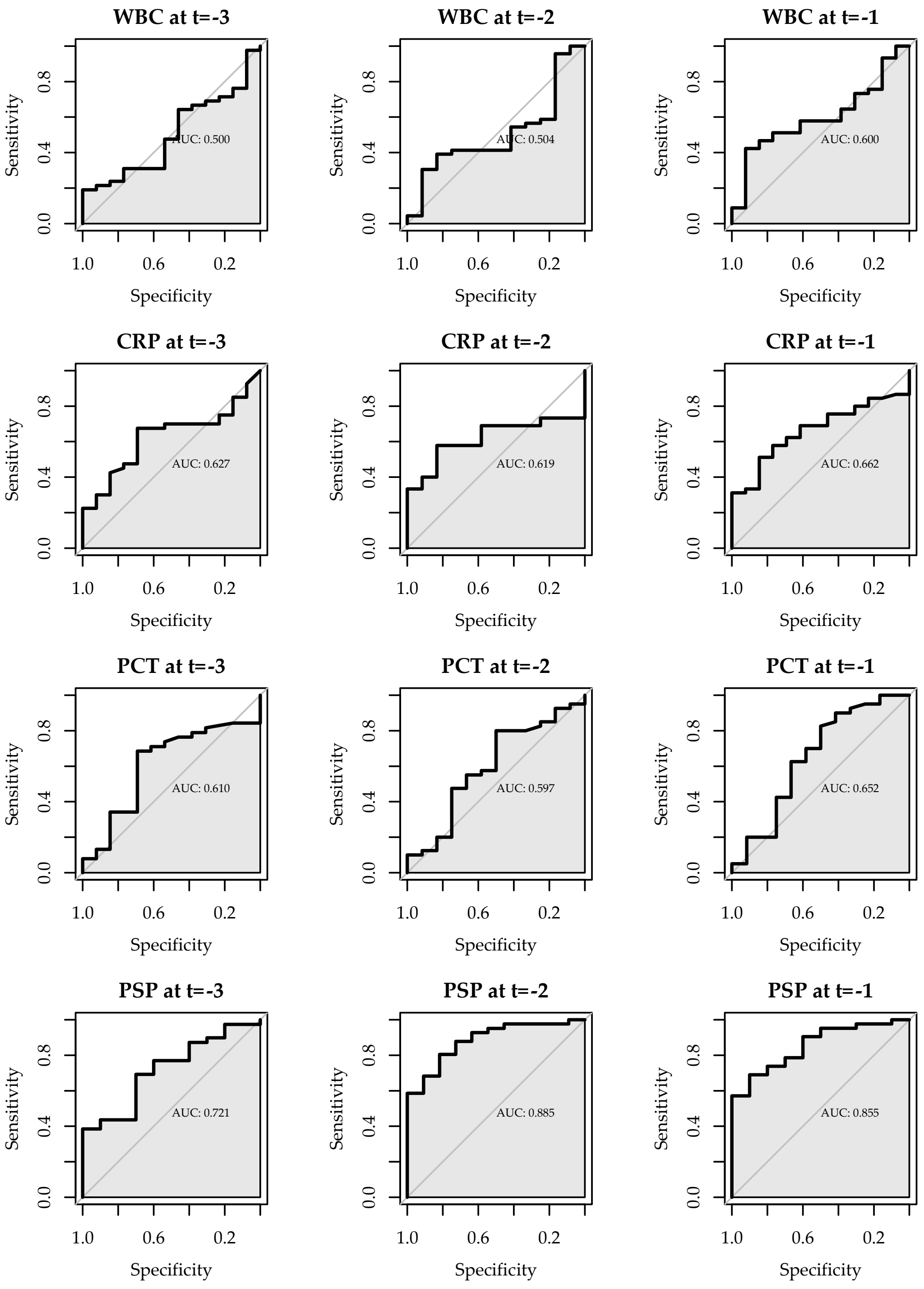

Figure S1. ROC-curve analysis with AUC for Sepsis-3. AUC of WBC at t-3, -2, -1 is between 0.5 and 0.6. AUC of CRP at t= -3, -2, -1 is between 0.62 and 0.66. AUC of PCT at t= -3, -2, -1 is between 0.59 and 0.65. The best performance is shown by PSP with an AUC of 0.72 (t= -3) 0.88 (t=-2) and 0.85 (t=.1) before the event of sepsis. The sensitivity at t= -3 is 0.7, t= -2 is 0.8 and t= -1 is 0.7. The specificity at t= -3 is 0.7, t= -2 is 0.81, t= -1 is 0.9.

Table S1. ROC-curve analysis for Sepsis-3.

Biomarker	Time (Days)	AUC	Threshold	Sensitivity	Specificity	Negative predictive value	Positive predictive value
WBC	-3	0.5	0.78	0.19	1	0.28	1
WBC	-2	0.5	0.8	0.39	0.83	0.26	0.9
WBC	-1	0.6	0.81	0.42	0.92	0.32	0.95
CRP	-3	0.63	0.73	0.68	0.69	0.41	0.87
CRP	-2	0.62	0.8	0.58	0.83	0.34	0.93
CRP	-1	0.66	0.8	0.51	0.85	0.33	0.92
PCT	-3	0.61	0.72	0.68	0.69	0.43	0.87
PCT	-2	0.60	0.74	0.8	0.5	0.43	0.84
PCT	-1	0.65	0.74	0.83	0.5	0.46	0.85
PSP	-3	0.72	0.73	0.69	0.7	0.37	0.9
PSP	-2	0.88	0.69	0.8	0.82	0.53	0.94
PSP	-1	0.85	0.8	0.69	0.9	0.41	0.97

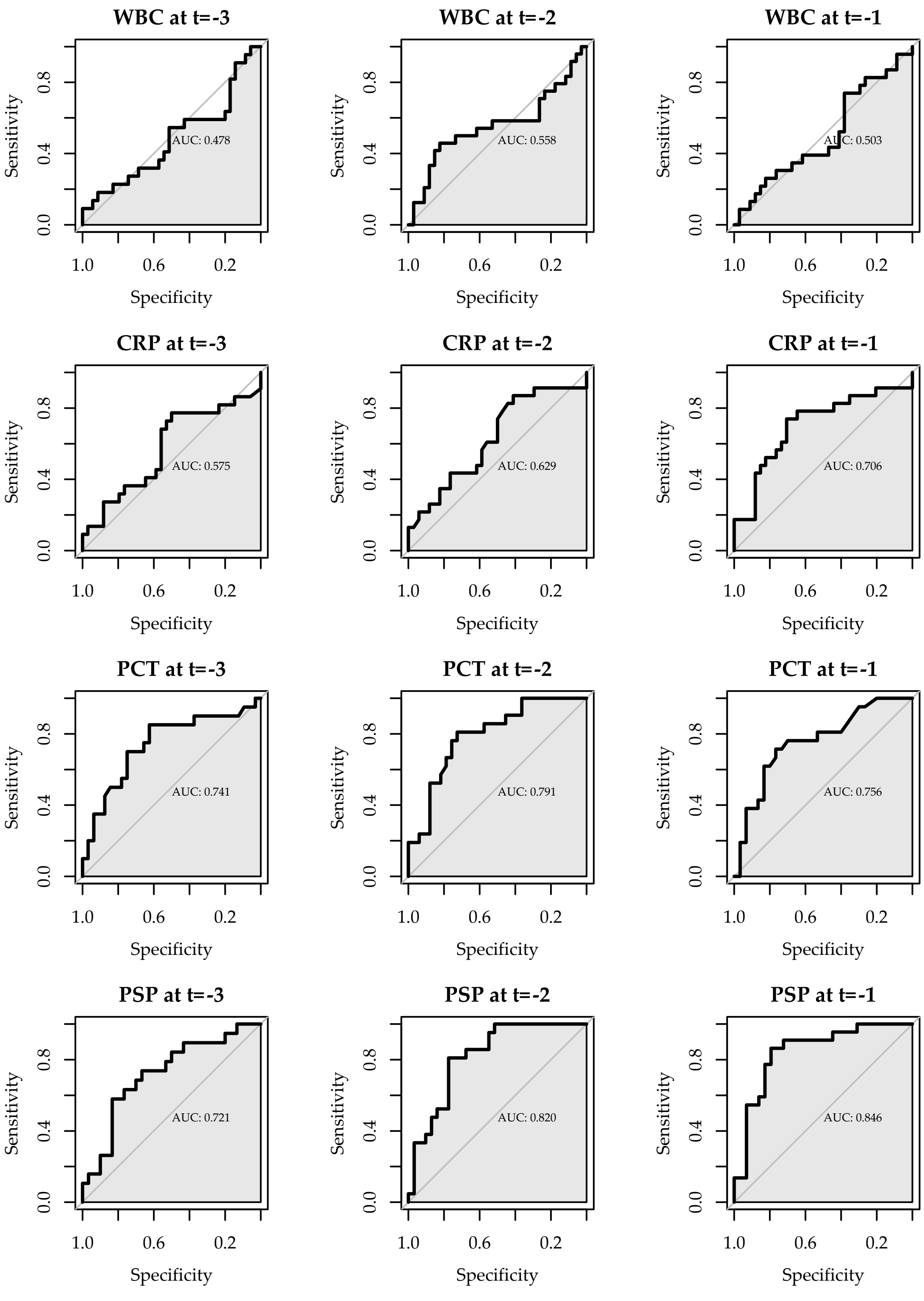

Figure S2. ROC-curve analysis with AUC for Sepsis ABA 2007. AUC of WBC at t-3, -2, -1 is between 0.5 and 0.55. AUC of CRP at t-3, -2, -1 is between 0.6 and 0.64. AUC of PCT at t-3, -2, -1 is between 0.6 and 0.62. The best performance is shown by PSP with an AUC of 0.64 (t= -3) 0.74 (t=-2) and 0.72 (t=.1) before the event of sepsis. The sensitivity at t= -3 is 0.6, t= -2 is 0.87 and t= -1 is 0.7. The specificity at t= -3 is 0.8, t= -2 is 0.56 and t= -1 is 0.75.

Table S2. ROC-curve analysis for Sepsis ABA 2007.

Biomarker	Time (Days)	AUC	Threshold	Sensitivity	Specificity	Negative predictive value	Positive predictive value
WBC	-3	0.52	0.58	0.42	0.8	0.51	0.74
WBC	-2	0.54	0.6	0.44	0.75	0.49	0.71
WBC	-1	0.56	0.59	0.47	0.73	0.51	0.70
CRP	-3	0.60	0.53	0.76	0.6	0.65	0.71
CRP	-2	0.64	0.57	0.71	0.65	0.6	0.75
CRP	-1	0.64	0.5	0.85	0.5	0.72	0.69
PCT	-3	0.60	0.55	0.78	0.52	0.63	0.69
PCT	-2	0.63	0.54	0.88	0.41	0.69	0.68
PCT	-1	0.61	0.54	0.67	0.61	0.58	0.69
PSP	-3	0.64	0.57	0.65	0.8	0.59	0.83
PSP	-2	0.74	0.51	0.88	0.57	0.75	0.76
PSP	-1	0.72	0.56	0.71	0.75	0.63	0.81

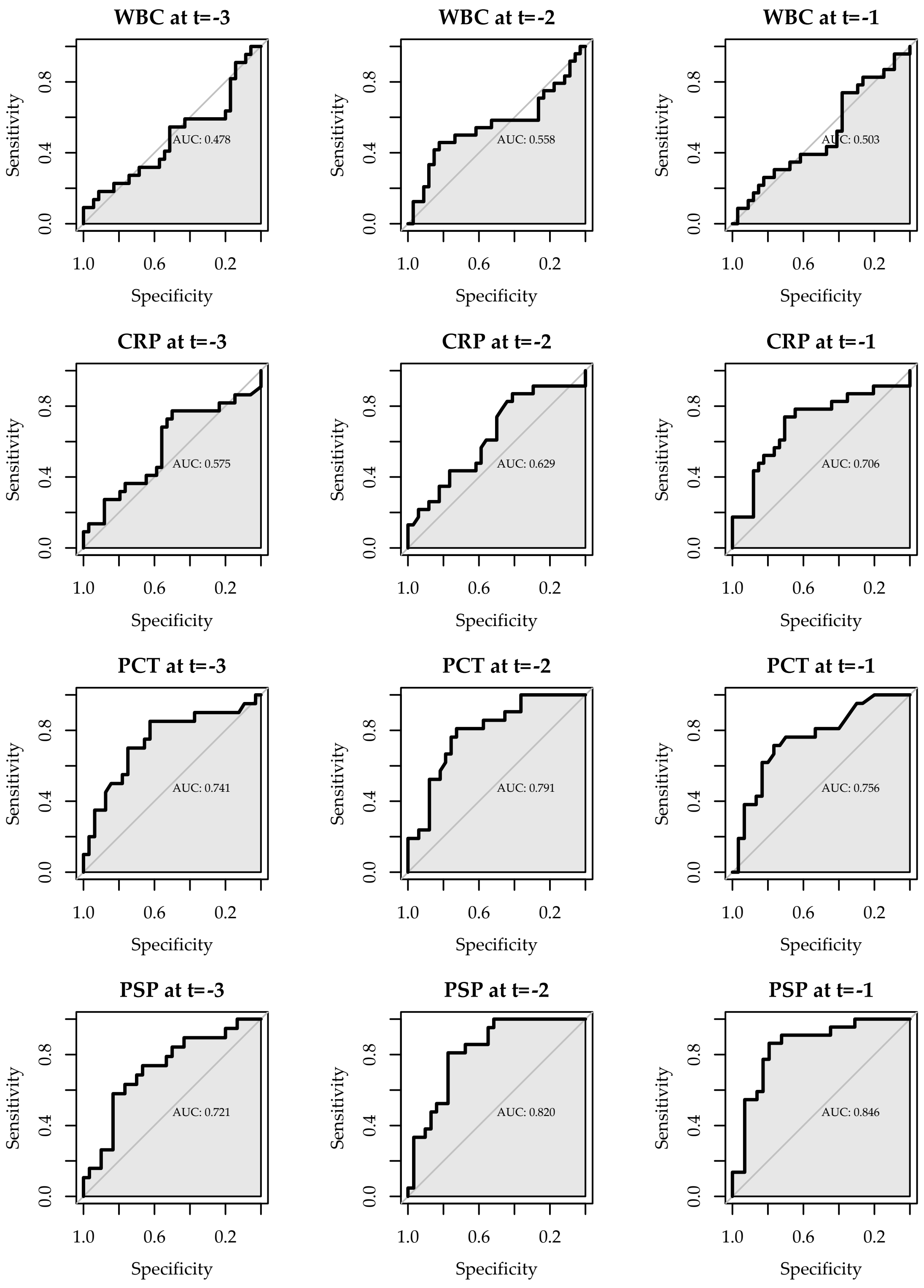

Figure S3. ROC-analysis with AUC for Sepsis Zurich Burn Centre. AUC of WBC at t= -3, -2, -1 is between 0.47 and 0.55. AUC of CRP at t=-3, -2, -1 is between 0.57 and 0.7. AUC of PCT at t=-3, -2, -1 is between 0.74 and 0.79. The best performance is shown by PSP with an AUC of 0.72 (t= -3) 0.82 (t=-2) and 0.84 (t=.1) before the event of sepsis. The sensitivity at t= -3 is 0.57, t= -2 is 0.81 and t= -1 is 0.85. The specificity at t= -3 is 0.83, t= -2 is 0.77 and t= -1 is 0.79.

Table S3. ROC-curve analysis for Sepsis Zurich Burn Centre.

Biomarker	Time (Days)	AUC	Threshold	Sensitivity	Specificity	Negative predictive value	Positive predictive value
WBC	-3	0.48	0.4	0.18	0.91	0.64	0.57
WBC	-2	0.56	0.43	0.46	0.82	0.68	0.65
WBC	-1	0.5	0.4	0.74	0.38	0.68	0.45
CRP	-3	0.57	0.36	0.77	0.5	0.77	0.5
CRP	-2	0.63	0.34	0.87	0.41	0.82	0.5
CRP	-1	0.71	0.4	0.74	0.71	0.8	0.63
PCT	-3	0.74	0.3	0.85	0.63	0.87	0.59
PCT	-2	0.79	0.33	0.81	0.73	0.86	0.65
PCT	-1	0.76	0.37	0.71	0.77	0.79	0.68
PSP	-3	0.72	0.36	0.58	0.83	0.76	0.69
PSP	-2	0.82	0.33	0.81	0.77	0.86	0.71
PSP	-1	0.85	0.33	0.86	0.79	0.88	0.76
